# Excessive positive response of model‐simulated land net primary production to climate changes over circumboreal forests

**DOI:** 10.1002/pei3.10025

**Published:** 2020-07-01

**Authors:** Shunsuke Tei, Atsuko Sugimoto

**Affiliations:** ^1^ Arctic Research Center Hokkaido University Sapporo Japan; ^2^ Graduate School of Environmental Science Hokkaido University Sapporo Japan; ^3^ Global Station for Arctic Research Global Institution for Collaborative Research and Education Hokkaido University Sapporo Japan; ^4^ North‐Eastern Federal University Yakutsk Russia

**Keywords:** climate change, earth system model, net primary production, normalized difference vegetation index, northern high‐latitude region, tree‐ring width index

## Abstract

Land carbon cycle components in an Earth system model (ESM) play a crucial role in the projections of forest ecosystem responses to climate/environmental changes. Evaluating models from the viewpoint of observations is essential for an improved understanding of model performance and for identifying uncertainties in their outputs. Herein, we evaluated the land net primary production (NPP) for circumboreal forests simulated with 10 ESMs in Phase 5 of the Coupled Model Intercomparison Project by comparisons with observation‐based indexes for forest productivity, namely, the composite version 3G of the normalized difference vegetation index (NDVI3g) and tree‐ring width index (RWI). These indexes show similar patterns in response to past climate change over the forests, i.e., a one‐year time lag response and smaller positive responses to past climate changes in comparison with the land NPP simulated by the ESMs. The latter showed overly positive responses to past temperature and/or precipitation changes in comparison with the NDVI3g and RWI. These results indicate that ESMs may overestimate the future forest NPP of circumboreal forests (particularly for inland dry regions, such as inner Alaska and Canada, and eastern Siberia, and for hotter, southern regions, such as central Europe) under the expected increases in both average global temperature and precipitation, which are common to all current ESMs.

## 1 INTRODUCTION

Land carbon cycle components in Earth system models (ESMs) play a crucial role in projecting the responses of forest ecosystems onto climate/environmental changes and global climate patterns (Ahlstrom, Schurgers, Arneth, & Smith, 2012; Garnaud, Sushama, & Arora, 2014; Jiang et al., 2015; Li, Lü, Liu, Gao, & Ao, 2015; Sato, Kobayashi, Iwahana, & Ohta, 2016; Shao, Zeng, Sakaguchi, Monson, & Zeng, 2013). However, these components should be tested properly to verify their capability of reproducing the heterogeneous response of ecosystems to climate changes of the past (Babst et al., 2014; Rammig et al., 2015; Rathgeber, Nicault, Kaplan, & Guiot, 2003; Tei, Sugimoto, Liang, et al., 2017; Tei, Sugimoto, Yonenobu, et al., 2017). Despite several modeling studies conducted with simulations of past and future forest dynamics of various forest types, the model performances are yet to be sufficiently evaluated (Hudiburg, Law, & Thornton, 2013; Running & Coughlan, 1998; Schaefer et al., 2012; Smith et al.., 2016).

Predictions from land carbon cycle components in ESMs, such as vegetation models, are primarily driven by photosynthesis with their parameterization being validated by temporal short‐term and spatial high‐resolution observations, such as eddy covariance carbon flux measurements (Mitchell, Beven, Freer, & Law, 2011). Accordingly, vegetation models can simulate short‐term forest carbon budgets with higher accuracy; however, annual and long‐term carbon budgets can also be insufficiently reproduced (Anderegg et al., 2015; Fatichi, Leuzinger, & Körner, 2014; Pappas, Mahecha, Frank, Babst, & Koutsoyiannis, 2017; Tei, Sugimoto, Liang, et al., 2017; Tei, Sugimoto, Yonenobu, et al., 2017; Thurner et al., 2017).

Remote sensing spectral vegetation indexes, such as the normalized difference vegetation index (NDVI; Tucker, 1979) and tree‐ring width index (RWI), may provide long‐term validation data for simulated plant productivity studies (Babst et al., 2018; Rammig et al., 20152019). This is because such datasets generally demonstrate a significant relationship with gross primary production, net primary production (NPP), and/or net ecosystem production of forest ecosystems (Dye et al., 2016; Klesse, Etzold, & Frank, 2016; Olofsson et al., 2008; Teets et al., 2018; Tei, Sugimoto, Kotani, et al., 2019) with adequate temporal resolution and sufficient duration. Tei, Sugimoto, Yonenobu, et al. (2017), Tei, Sugimoto, Liang, et al. (2017) recently reported contrary responses of circumboreal forests to warming deduced from the RWI and a spatially explicit individual‐based dynamic global vegetation model (SEIB‐DGVM; Sato et al., 2016). These studies indicated that the DGVM could not reproduce the dominant negative response of tree growth to warming in continentally dry and relatively warm regions, such as inner Alaska and Canada, the central part of Europe, and eastern Siberia, deduced by the RWI.

Previous research on tree‐rings (Barber, Juday, & Finney, 2000; Girardin et al., 2016; Tei, Nagai, & Sugimoto, 2019; Tei, Sugimoto, Kotani, et al., 2019; Tei, Sugimoto, Yonenobu, et al., 2017; Tei, Yonenobu, Sugimoto, Ohta, & Maximov, 2015; Walker, Michelle, & Johnstone, 2015) indicated a one‐year time lag in the tree growth response to climate variabilities in these regions, which is regarded as an adaptation of these ecosystems to severely water‐stressed environments (Chapin, Schultz, & Mooney, 1990; Genet, Breda, & Dufrene, 2010; McDowell et al., 2008; Wiley & Helliker, 2012). Tei and Sugimoto (2018) determined that this is a common phenomenon observable by satellite imagery. However, such a time lag response could not be reproduced by the DGVM used by Tei, Sugimoto, Yonenobu, et al. (2017), Tei, Sugimoto, Liang, et al. (2017).

It remains unclear whether the above discrepancies in forest responses to climate change between the observation‐based indexes (e.g. composite version 3G of the NDVI (NDVI3g) and RWI) and DGVM output is a feature of the DGVM used by Tei, Sugimoto, Yonenobu, et al. (2017), Tei, Sugimoto, Liang, et al. (2017) or a more generalized feature for all models. Klesse et al. (2018) reported that most of the current DGVM generations strongly overestimate the temperature sensitivity of cold environments over European forests. In addition, mismatches in correlations with summer climate variables between DGVMs and RWI (Babst et al., 2013) and those of lag effects of previous‐year summer climates (Zhang et al., 2018) have also been reported for European forests. However, it is still unclear whether these discrepancies may be observed for other ecosystems beyond European forests. Therefore, we compared observation‐based indexes for forest productivity, namely, the NDVI3g and RWI, with the land NPP simulated by land carbon cycle components of 10 ESMs involved in Phase 5 of the Coupled Model Intercomparison Project (CMIP5) (as opposed to a single vegetation model). We performed this comparison to gain a better understanding of model performance and identify uncertainties in their output over circumboreal forests, similar to the aforementioned discrepancies in previous studies (Tei, Nagai, et al., 2019; Tei & Sugimoto, 2018; Tei, Sugimoto, Liang, et al., 2017; Tei, Sugimoto, Yonenobu, et al., 2017).

Circumboreal forest ecosystems cover 22% of the Earth's terrestrial surface and account for 12% of the global NPP, thereby playing an important role in attenuating recent global warming through photosynthesis (Chapin et al., 2005; Kimball et al., 2006; McGuire et al., 2009). Under the strong warming trend in high latitudinal areas, i.e., Arctic amplification (Serreze & Barry, 2011), drastic changes, such as permafrost degradation (Lemke et al., 2007), changes in the tree line location (Frost & Epstein, 2014; Tchebakova, Parfenova, & Soja, 2009), shifts in tree growth patterns (Tei, Sugimoto, Liang, et al., 2017; Tei, Sugimoto, Yonenobu, et al., 2017), and forest/tree decay (Allen, Breshears, & McDowell, 2015; Tei, Morozumi, et al., 2019; Tei, Sugimoto, Yonenobu, Kotani, & Maximov, 2019), have all been observed and are processes that would be expected to continue in circumboreal forest ecosystems. However, significant uncertainty remains about the magnitude and location of these spatial variations, which serve to alter the ecosystems’ ability to behave as a carbon sink (i.e., the amount of carbon dioxide absorbed from the atmosphere would be altered) (Ciais et al., 2010; Goodale et al., 2002). Therefore, understanding spatial variations in the response of forest ecosystems to climate change in the northern high‐latitude regions is crucial for accurate projection of the terrestrial carbon cycle and global climate.

## 2 MATERIALS AND METHODS

### 2.1 Satellite vegetation greenness datasets

We employed the bimonthly maximum NDVI3g dataset, which is the latest dataset released by the Global Inventory Modeling and Mapping Studies group (Pinzon & Tucker, 2014; Tucker et al., 2005), and covers the period of 1981–2015 with a native spatial resolution of 0.083°. The NDVI product is an indicator of photosynthetic activity (Myneni, Ramakrishna, Nemani, & Running, 1997) and interannual variability in tree growth over circumboreal ecosystems (Berner, Beck, Bunn, Lloyd, & Goetz, 2011; Bunn et al., 2013), computed as the difference between the near‐infrared and red reflectance of the land surface normalized by the sum of the reflectances. NDVI3g comprises recently revised data of its previous version, NDVI version G (NDVIg), which specifically aims to improve the data quality for high‐latitude regions (Zhu et al., 2013). Explicit corrections of the effects of orbital drift and stratospheric aerosols from volcanic eruptions have also been applied (Tucker et al., 2005). Therefore, it is suitable for studying changes in vegetation activities in northern high‐latitude regions (Guay et al., 2014; Xu et al., 2013).

NDVI3g average summer values were used for the analyses. In consideration of the different lengths of the plant‐growing season (e.g., Lund et al., 2010), the definition of the summer season varied depending on the latitude. Specifically, only one month (July) was defined as summer for the sites to the north of the Arctic circle (~67° N), whereas three months (June, July, and August) were considered as summer for the sites located between 50° N and 67° N.

### 2.2 Tree‐ring datasets

Raw tree‐ring width chronologies were collected from 600 sites of the International Tree‐Ring Data Bank (http://www.ncdc.noaa.gov/paleo/treering.html; accessed during spring–winter 2014). The following criteria were employed for sample site selection: (a) sites were located farther north than 50°N to represent the southern limit of boreal forests; (b) the sites had an elevation lower than 2000 m above sea level to avoid excessive sampling from temperature‐sensitive trees due to the cold environment in high altitude areas; and (c) sites had a chronology ending after 1990 to better represent the effects of recent global warming (Table S1). In addition, we incorporated six published chronologies from our previous works (Tei, Sugimoto, Liang, et al., 2017; Tei, Sugimoto, Yonenobu, Ohta, & Maximov, 2014) (Table S2).

We generated a standard RWI for each site using the ARSTAN software (Cook, 1985). The standard RWI was developed by first detrending the growth trends associated with tree age and natural disturbances in a raw time series, which were derived from the single dominant tree species in each site by cubic smoothing splines and subsequently averaging the standardized ring widths among the samples (Cook, 1985). Therefore, we applied spline fitting to standardize the chronologies at all sites, which is regarded as being a relatively site‐insensitive method (Cook, 1985; Cook & Kairiukstis, 1990); thus, this is in line with our approach involving various forest ecosystem types (Table S1 and S2). Spline fits with a 50％ frequency cutoff at a wavelength of 128 years were applied to all raw time series.

RWI quality was assessed by the expressed population signal, which is a measure of how well a finite sample of tree‐ring data represent an infinite population chronology. The signal value 0.85 was considered a threshold of acceptable statistical quality for dendrochronological analyses (Wigley, Briffa, & Jones, 1984). The expressed population signal values for 554 of the 606 sites were higher than 0.85 after 1950, showing a high degree of common variability between individual trees (Tables S1 and S2). Therefore, for subsequent analyses, we used the RWI of these 554 sites.

### 2.3 Earth system models

We employed 10 ESMs involved in CMIP5 (Table 1) and selected one historical simulation from each ESM (i.e. a total of 10 historical simulations) for our model performance evaluations according to Jiang et al. (2015). These authors selected one simulation from each modeling group with the global sum of vegetation carbon closest to that of the observational data, except for CESM1 (from the modeling group Community Earth System Model Contributors), IPSL‐CM5A‐MR (from L’Institute Pierre‐Simon La Place), and NorESM1‐ME (from Norwegian Climate Center), which were recommended by the respective modeling groups. The ESMs and their major differences in simulating the land NPP are described in Table 1. We downloaded the monthly land NPP for the historical simulations by these models (http://www.ipcc‐data.org/sim/gcm_monthly/AR5/index.html). Where multiple ensemble members were available from a single historical simulation, we calculated the ensemble mean for these members. Finally, we calculated the annual sum of the monthly land NPP. These parameters were all used for further analyses.

**TABLE 1:** Summary of CMIP5 ESMs and their characteristics for the land carbon cycle (modified by Jiang et al., 2015)

Model name	Model name ID	Modeling group	Land carbon cycle components	Number of PFTs	Dynamic vegetation	Nitrogen cycle	Original resolution (lat. ×lon.)	Reference
BCC_CSM1.1	BCC	Beijing Climate Center (BCC), ChainaMeteorological Administration	BCC Atmospheric and Vegetation Interaction Model, version 1.0 (BCC_AVIM1.0)	15	×	×	2.81°×2.81°	Ji, Huang, and Li (2008); Wu et al. (2013)
BNU‐ESM	BNU	College of Global Change and Earth SystemScience, Beijing Normal University (BNU)	Common Land Model (CoLM)and BNU DGVM [based onLund‐Potsdam‐Jene model (LPJ)]	10	〇	×	2.81°×2.81°	Dai et al. (2003, 2004); Ji et al. (2014)
CanESM2	CanESM2	Canadian Center for Climate Modeling andAnalysis	Canadian Land Surface Scheme, version 2.7 (CLASS2.7) and CTEM, version 1 (CTEM1)	9	×	×	2.81°×2.81°	Arora and Boer (2010)
CESM1	CESM1	Community Earth System ModelContributors	Community Land Model, version4 (CLM4)	15	×	〇	0.94°×1.25°	Lawrence et al. (2011); Thornon and Zimmermann (2007); Thornton, Lamarque, Rosenbloom, and Mahowald (2007, 2009)
GFDL‐ESM2G	GFDL	Geophysical Fluid Dynamics Laboratory	Land Model, version 3 (LM3)	5	〇	×	1.99°×2.48°	Dunne et al. (2013); Shevliakiova et al. (2009)
HadGEM2‐ES	Had	Met Office Hadley Center	TRIFFID	5	〇	×	1.24°×1.88°	Collins et al. (2011); Cox (2001); Jones et al. (2011); Martin et al. (2011)
IPSL‐CM5A‐MR	IPSL	L'Institute Pierre‐Simon Laplace	Land Surface Model (LSM), version 1.0 ORCHIDEE	12	×	×	2.26°×2.50°	Dufresne et al. (2013); Krinner et al. (2005)
MIROC‐ESM	MIROC	Japan Agency for Marine‐Earth science and Technology, Atmosphere and Ocean Research Institute (The University of Tokyo), and National Institute for Environmental Studies	Minimal Advanced Treatments of Surface Interaction and Runoff (MATSIRO) and SpatiallyExplicit Individual‐Based DGVM(SEIB‐DGVM)	13	〇	×	2.81°×2.81°	Sato, Itoh, and Kohyama (2007); Watanabe et al. (2011)
MPI‐ESM‐MR	MPI	Max Planck Institute for Meteorology	JSBACH	12	〇	×	1.88°×1.88°	Brovkin, Raddatz, Reick, Claussen, and Gayler (2009); Raddatz et al. (2007); Reick, Raddatz, Brovkin, and Gayler (2013)
NorESM1‐ME	Nor	Norwegian Climate Center	CLM4	15	×	〇	1.88°×2.50°	Tjiputra et al. (2013)

### 2.4 Data re‐sampling for a consistent grid

There was a substantial mismatch in spatial scale between the RWI, NDVI3g, and ESM‐simulated land NPP. The majority of the tree cores were collected over an area around one hectare, whereas each NDVI3g grid cell covered ~64 km^2^ and each ESM grid cell covered 10,000–30,000 km^2^. Therefore, in this study, all ESMs were re‐sampled into a 2.81° × 2.81° consistent grid, which was the largest grid used in the model. NDVI3g grid cells were then averaged to the consistent grid. RWIs from multiple sites were averaged if they occurred within the consistent grid. In this way, all ESMs and NDVI3g represented a spatial scale of the same size, and the RWI was as close to that spatial scale as possible, allowing for better aggregation. Aggregating the RWI and NDVI3g over this broader area ensured a better representation of the conditions simulated by the ESMs.

### 2.5 Climate variables

For the comparison of RWI and NDVI3g, we used gridded monthly near‐surface average temperature and total precipitation datasets (1901–2016; 0.5° longitude by 0.5° latitude) (CRU TS 4.01.; Harris, Jones, Osborn, & Lister, 2014) to extract monthly climatic records and calculate spatial average values from the RWI and NDVI3g 2.81° × 2.81° grids (hereafter referred to as “grids”). Monthly temperature (variable *tas*) and precipitation (variable *pr*) were calculated from each ESM, were downloaded, and used for comparisons with the land NPP simulated by the ESMs after calculating the spatial average values of the grids. With the aim of determining the main climatic driver for the simulated land NPP, climate variables for the NPP simulation in each ESM, rather than CRU climate records, were used for the comparison. Similar to the NDVI3g data, we used the average summer temperature and precipitation values for our analyses, with the one month and three months summer definitions for higher latitudes (July for Arctic circle (~67°N) and June–July–August for the sites located between 50°N and 67°N).

### 2.6 Correlation function analyses

Correlation function analyses were performed using the R software (R Core Team, 2017) to identify relationships of summer temperatures and precipitation with the RWI, NDVI3g, and ESM‐simulated land NPP. We applied a significance level of *p* < .05. The analysis period for the NDVI3g and simulated land NPP time series was from 1982 to 2006 (25 years) with overlaps. Since many grid‐average RWI chronologies ended in the 1990s, the analysis period for the RWI was determined to be 25 years, counted retroactively from the last year of each grid‐average RWI chronology.

To determine correlations with climate variables, we considered not only the climate conditions of the current summer but also those of previous summers. Previous tree‐ring and satellite image studies showed that climate conditions during the previous year substantially affected tree radial growth, especially in arid regions (Babst et al., 2013; Tei & Sugimoto, 2018; Tei, Sugimoto, Yonenobu, Hoshino, & Maximov, 2013; Tei, Sugimoto, Yonenobu, Yamazaki, & Maximov, 2013; Tei et al., 2015; Zhang et al., 2018). Correlation coefficients of summer temperature and precipitation were calculated based on these data, both from previous and current years and for each grid. In some analyses, the highest correlation coefficients between them were used to identify the most relevant climate variable to the RWI, NDVI3g, and ESM‐simulated land NPP, including the time lag effect.

**FIGURE 1:**
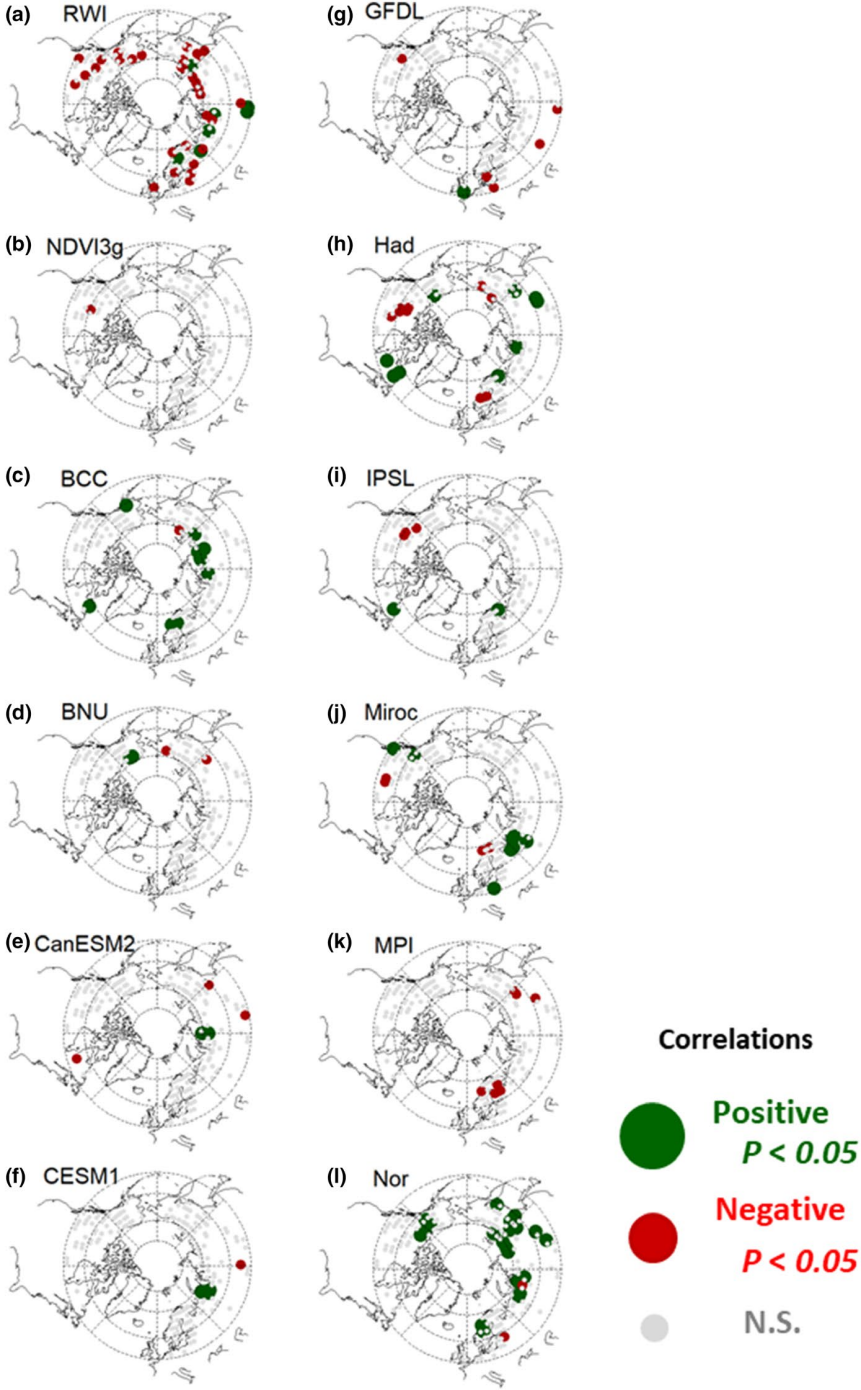
Correlation coefficients of (a) RWI; (b) NDVI3g; and (c–l) land NPP by ESMs with previous summer temperature during the last 25 years of chronology for the RWI and during 1982–2006 for the NDVI3g and NPP by ESMs

**FIGURE 2:**
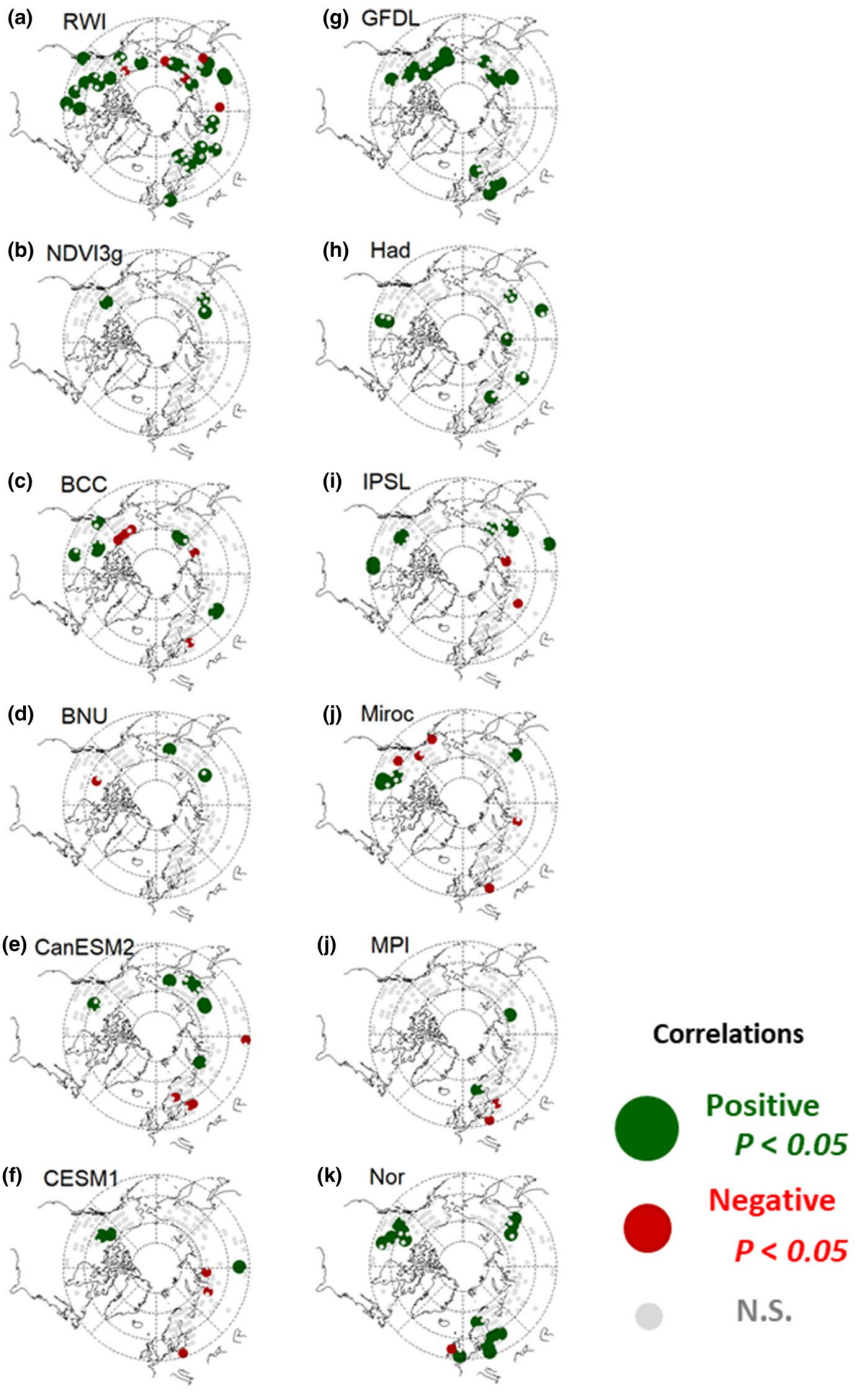
Correlation coefficients of (a) RWI; (b) NDVI3g; and (c–l) land NPP by ESMs with previous summer precipitation during the last 25 years of chronology for the RWI and during 1982–2006 for the NDVI3g and NPP by ESMs

**FIGURE 3:**
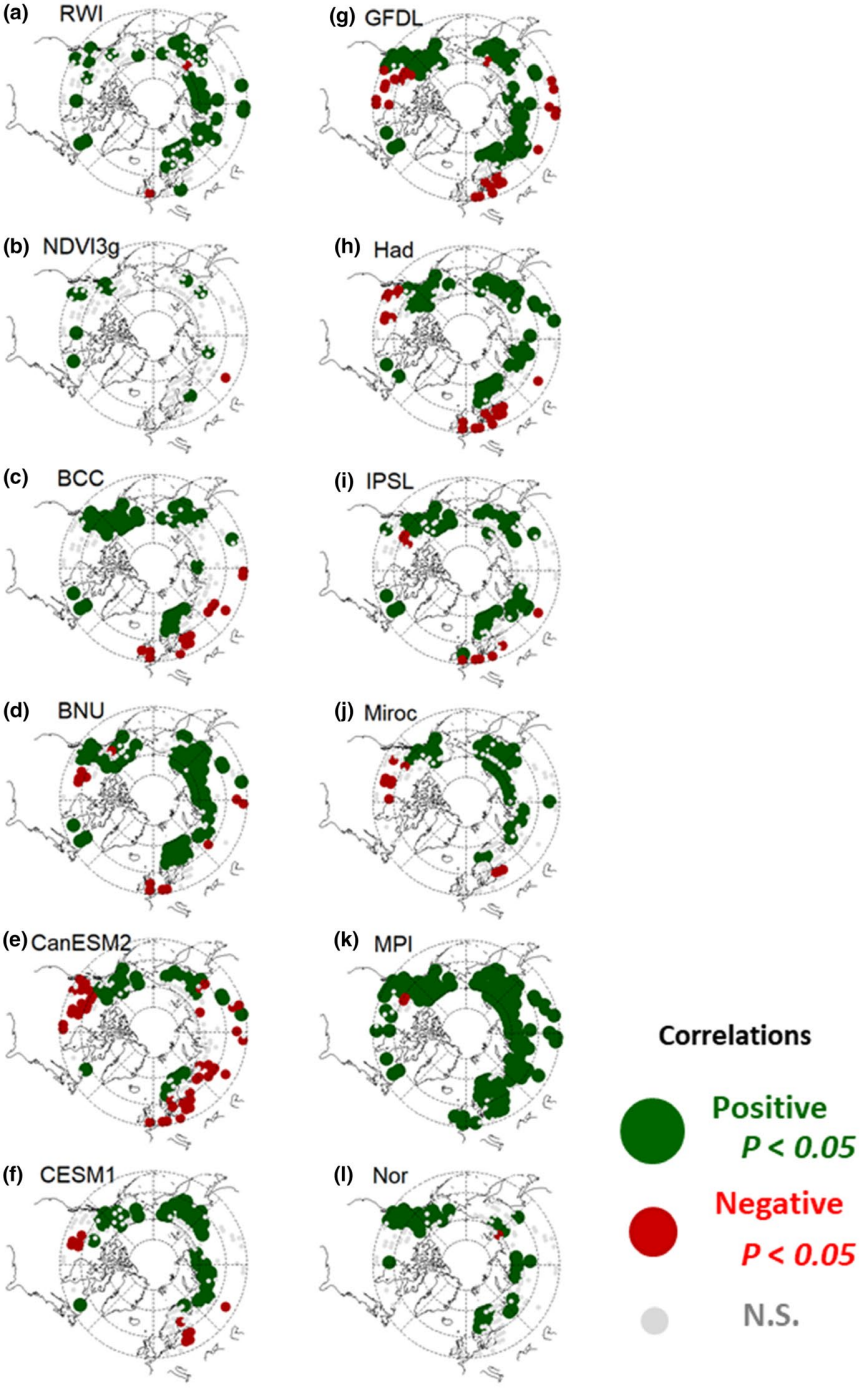
Correlation coefficients of (a) RWI; (b) NDVI3g; and (c–l) land NPP by ESMs with current summer temperature during the last 25 years of chronology for the RWI and during 1982–2006 for the NDVI3g and NPP by ESMs

**FIGURE 4:**
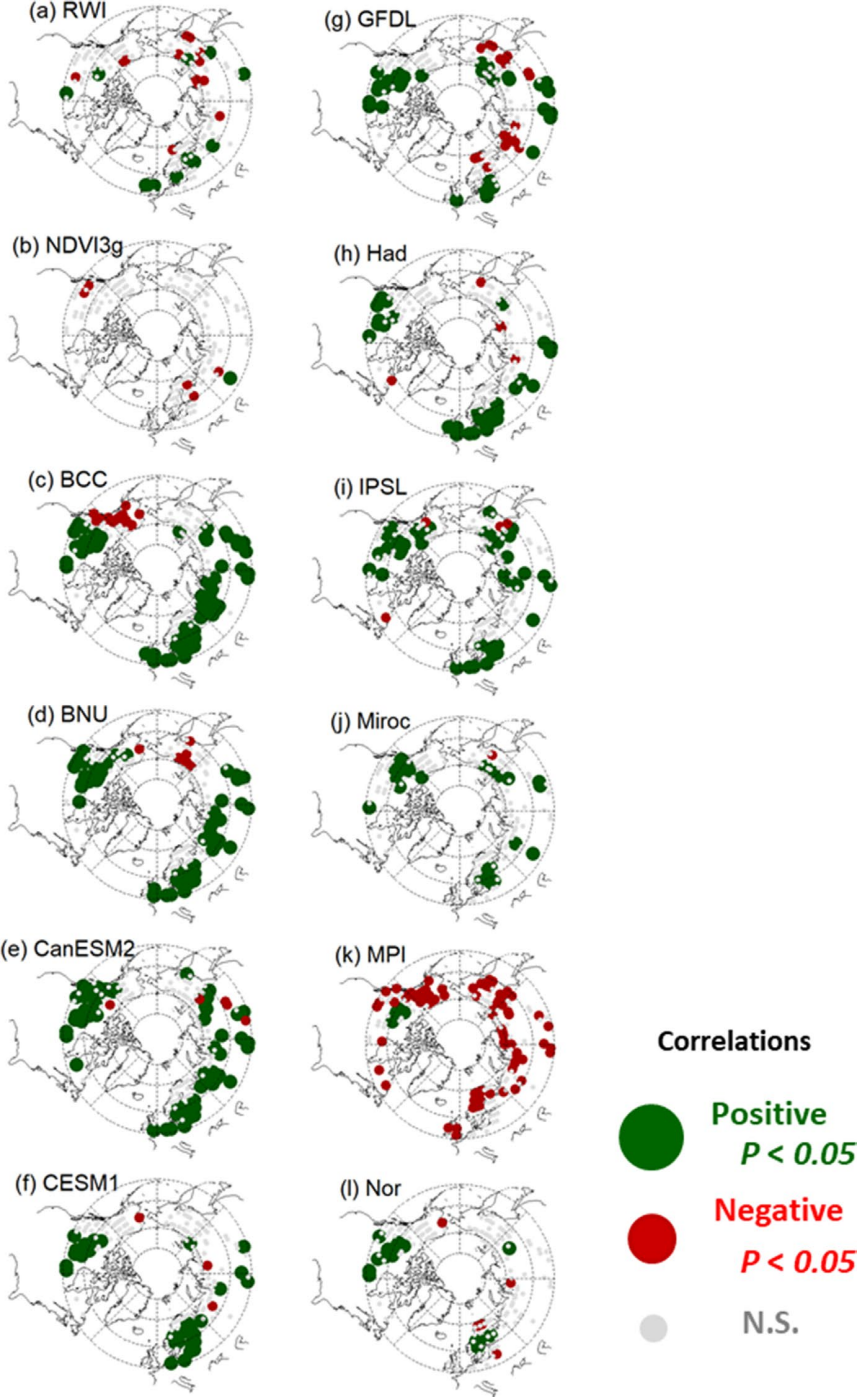
Correlation coefficients of (a) RWI; (b) NDVI3g; and (c–l) land NPP by ESMs with current summer precipitation during the last 25 years of chronology for the RWI and during 1982–2006 for the NDVI3g and NPP by ESMs

**FIGURE 5:**
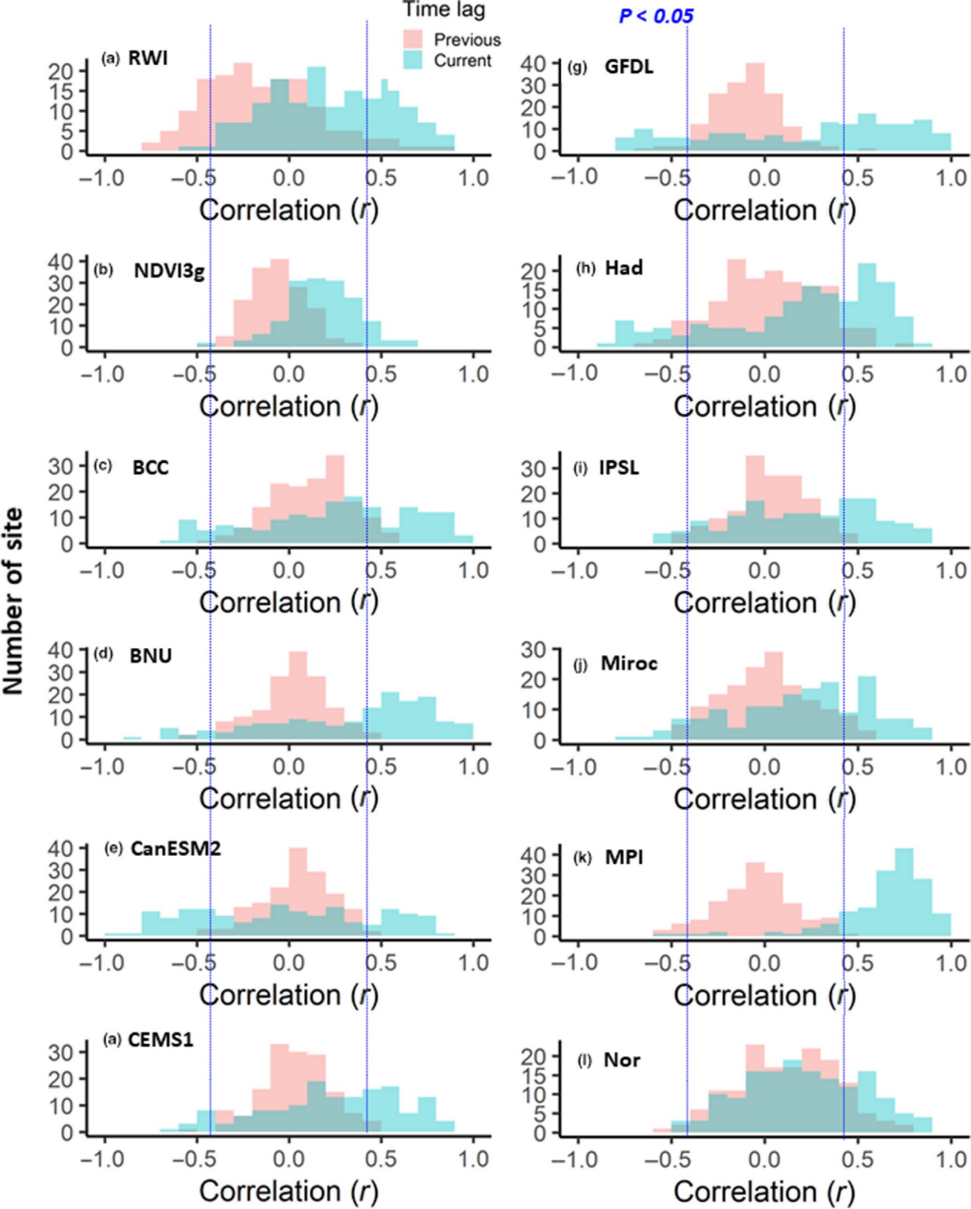
Histogram for correlations of (a) RWI; (b) NDVI3g; and (c–l) land NPP by ESMs with summer temperature for the previous and current year

**FIGURE 6:**
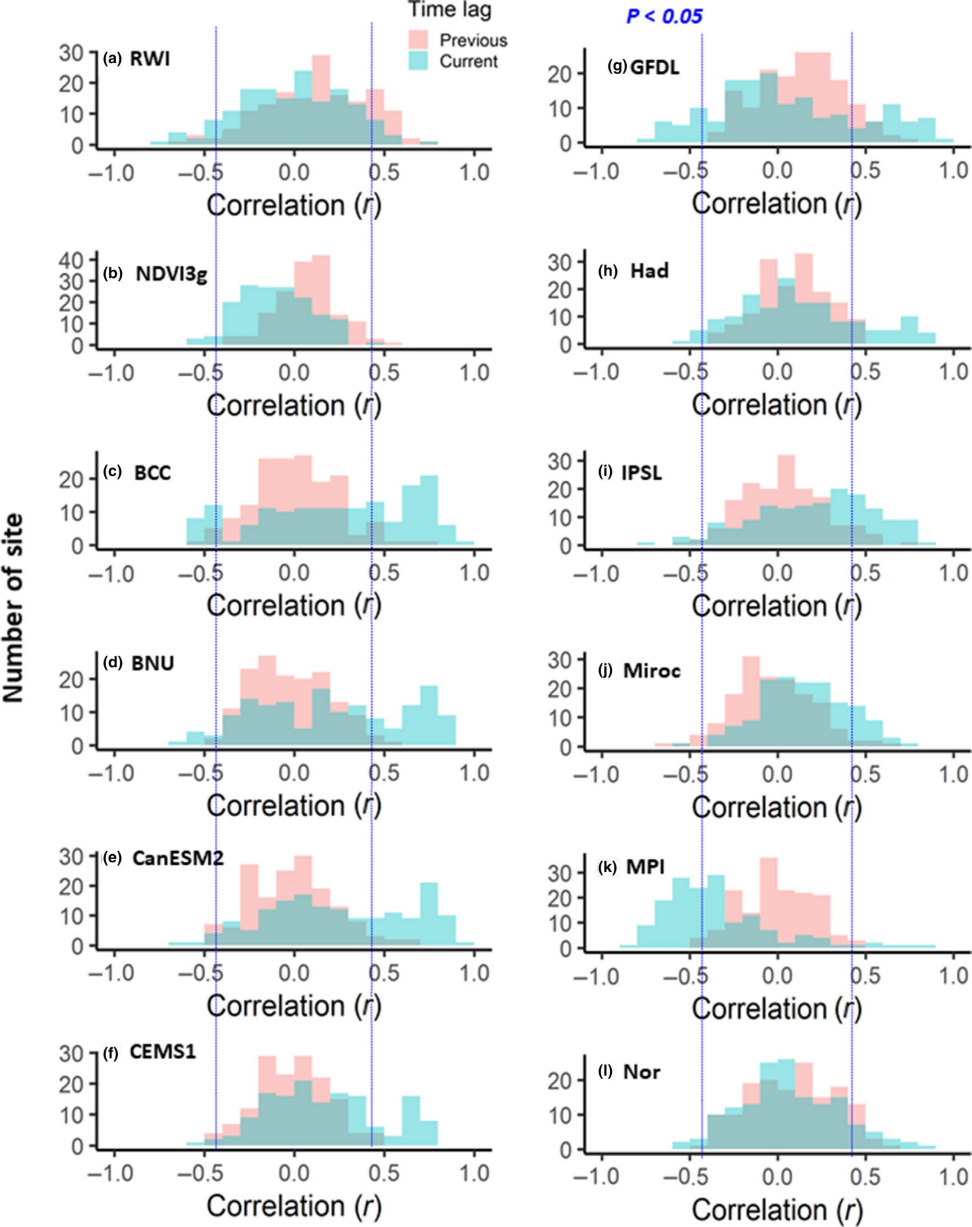
Histogram for correlations of (a) RWI; (b) NDVI3g; and (c–l) land NPP by ESMs with summer precipitation for the previous and current year

Since 25 years was a relatively short time span for detecting the relationship with climate variables, a similar analysis was performed on data from 1950 and later for the RWI and ESM‐simulated land NPP. Although the significance level of the correlations changed slightly, the duration of the correlation analysis did not significantly affect the spatial distribution of the correlation (Figures 1, 2, 3, 4, and Figures S1–S4) or the shape of its histogram (Figures 5, 6 and Figures S5 and S6), indicating the robustness of our analytical results for the RWI, NDVI3g, and ESM‐simulated land NPP responses to changes in summer temperature and precipitation over the past 25 years.

### 2.7 Density plot

Density plots for the correlations of the RWI, NDVI3g, and ESM‐simulated land NPP with climate variables were generated using the R software and calculated based on meteorological data for temperature and precipitation in both the previous and current year for each grid. The highest correlation coefficients for previous, current, and both year data were used separately for the density plots, which were variations of histograms that used kernel smoothing to plot smoother value distributions by eliminating noise. This approach was helpful in visualizing the correlation distribution over the continuous time interval. The peaks of the density plots occurred where the values were concentrated.

## 3 RESULTS

### 3.1 Spatial correlation patterns of the RWI, NDVI3g, and simulated land NPP with climate variables

Figures 1 and 2 show the spatial variations in past relationships of the RWI, NDVI3g, and ESM‐simulated land NPP with previous‐year summer temperature and precipitation, respectively. The RWI had a larger areal extent, with significant negative and positive correlations with summer temperature and precipitation, respectively, compared with the NDVI3g and land NPP. Among the RWI grids, 19.6% and 17.7% showed significant negative and positive correlations with summer temperature and precipitation, respectively (Table 2). These significant correlations were dominant for inner Alaska and Canada, central Europe, and eastern Siberia (Figures 1a and 2a). The NDVI3g and some ESMs also indicated negative correlations with previous‐year summer temperature in the latter three regions, but not within inner Alaska (Figure 1).

**TABLE 2:** Number and percentage of grids with positive or negative responses to temperature and precipitation of the previous summer; the correlation coefficients were calculated based on meteorological data from the previous year for each grid

	Positive_t		Negative_t		Positive_p		Negative_p	
	Number of grid	Percentage (%)	Number of grid	Percentage (%)	Number of grid	Percentage (%)	Number of grid	Percentage (%)
RWI	7	4.4	31	19.6	28	17.7	5	3.1
NDVI3g	0	0	1	0.6	3	1.8	0	0
BCC	11	7	1	0.6	9	5.6	6	3.7
BNU	2	1.3	2	1.3	2	1.3	1	0.6
CanESM2	2	1.3	3	1.9	7	4.4	4	2.5
CESM1	3	2	1	0.7	3	2	3	2
GFDL	1	0.6	5	3.2	19	12	0	0
Had	9	6	10	6.6	7	4.6	0	0
IPSL	2	1.3	3	1.9	9	5.8	3	1.9
MIROC	8	5.2	4	2.5	5	3.2	5	3.2
MPI	0	0	8	5	2	1.2	2	1.2
Nor	21	13.8	2	1.3	15	9.8	1	0.6

Note

Positive_t and Negative_t indicate significant (*p* < .05) positive and negative correlations for summer temperature, respectively. Positive_p and Negative_p indicate significant (*p* < .05) positive and negative correlations for summer precipitation, respectively.

Figures 3 and 4 show the identical spatial variations as Figures 1 and 2, but with current‐year data. Compared to the previous‐year data, the NDVI3g and land NPP had a larger areal extent, with significant and mostly positive correlations. The percentage of grids showing a significant positive correlation with summer temperature was 6.9%–86.7%, while the percentage of grids showing a significant negative correlation was 0.6%–27.8% (Table 3). Excluding the ESM MPI that had larger grids with significant negative correlations with the current‐year summer precipitation (48.7% of total grids) than the other ESMs, the ESM‐simulated land NPP tended to show a significant positive correlation with summer precipitation. The percentage of grids having a significant positive correlation with summer precipitation was 0.6%–40.5%, while the percentage of grids showing a significant negative correlation was 0.6%–12.6%.

**TABLE 3:** Number and percentage of grids with positive or negative responses to temperature and precipitation of the current summer; the correlation coefficients were calculated based on meteorological data from the current year for each grid

	Positive_t		Negative_t		Positive_p		Negative_p	
	Number of grid	Percentage (%)	Number of grid	Percentage (%)	Number of grid	Percentage (%)	Number of grid	Percentage (%)
RWI	51	32.2	2	1.2	11	6.9	13	8.2
NDVI3g	11	6.9	1	0.6	1	0.6	5	3.1
BCC	57	36	15	9.4	64	40.5	16	10.1
BNU	84	55.2	12	7.8	51	33.5	8	5.2
CanESM2	36	22.7	44	27.8	59	37.3	5	3.1
CESM1	50	33.5	11	7.3	33	22.1	3	2
GFDL	74	46.8	27	17	34	21.5	20	12.6
Had	60	40	20	13.3	31	20.6	5	3.3
IPSL	57	36.7	9	5.8	46	29.6	4	2.5
MIROC	47	30.5	10	6.4	24	15.5	1	0.6
MPI	137	86.7	2	1.2	5	3.1	77	48.7
Nor	44	28.9	1	0.6	16	10.5	5	3.2

Note

Positive_t and Negative_t indicate significant (*p* < .05) positive and negative correlations for summer temperature, respectively, whereas Positive_p and Negative_p indicate significant (*p* < .05) positive and negative correlations for summer precipitation, respectively.

The spatial distribution of correlations with current climate variables among the RWI, NDVI3g, and ESMs varied. The RWI, NDVI3g, and most ESMs, except for ESM Had, CanESM2, CESM1, and Miroc, showed significant correlations with the current summer temperature in coastal regions over Canada and grids located farther north than 60° N over Eurasia, with mostly positive correlations (Figure 3). In contrast, ESM Had, CanESM2, CESM1, and Miroc indicated either significant negative correlations or no correlation with the current‐year summer temperature in the coastal regions over Canada. For the current‐year summer precipitation, the RWI and most ESMs (except ESM MPI and Nor) showed dominant and significant positive correlations in grids located farther south than 60°N over Europe (Figure 4). Otherwise, most of the significant correlations with the current‐year summer precipitation were negative for the NDVI3g and ESM MPI.

When the highest correlation of the RWI, NDVI3g, and land NPP with the summer temperature and precipitation between the previous and current year was applied, the effect of temperature on ESMs was significantly more positive for grids where temperature was more correlated than precipitation; however, the negative effect was relatively well observed for the RWI, where 48 and 21 grids, respectively, showed significant positive and negative correlations with summer temperature (Table 4). In grids where precipitation was more strongly correlated than temperature, the effect of precipitation on the RWI and most ESMs was significantly more positive; however, the positive and negative effects were nearly equal for the NDVI3g and ESM MPI.

**TABLE 4:** Intensity of effects for summer temperature and precipitation for the RWI, NDVI3g, and ESMs

	Grids with more significant correlation for temperature than for precipitation		Grids with more significant correlation for precipitation than for temperature	
	Positive_t	Negative_t	Positive_p	Negative_p
RWI	48	21	26	9
NDVI3g	9	1	4	4
BCC	62	1	67	3
BNU	81	2	45	1
CanESM2	36	11	50	3
CESM1	50	2	33	2
GFDL	69	11	37	6
Had	60	13	31	2
IPSL	54	0	49	3
MIROC	49	11	23	4
MPI	131	1	5	6
Nor	58	3	23	4

Note

Positive_t and Negative_t indicate the number of grids with more significant positive and negative correlations for summer temperature, respectively. Positive_p and Negative_p indicate the number of grids with more significant positive and negative correlations for summer precipitation, respectively.

### 3.2 Histogram for correlations of the RWI, NDVI3g, and land NPP with climate variables

Figure 5 shows the histograms for the correlations of the RWI, NDVI3g, and ESM‐simulated land NPP with the summer temperature for previous‐ and current‐year data. The RWI and NDVI3g had relatively similar histograms, in that correlations with the current‐ and previous‐year summer temperatures were more positive and negative, respectively, especially for the RWI. Among the RWI 2.81° × 2.81° grids, 32.2% and 19.6% showed significant positive and negative correlations with the current‐ and previous‐year summer temperature, respectively (Tables 2 and 3). Correlations of the RWI and land NPP with the current‐year summer temperature were more positive than those of the NDVI3g, indicating that the percentages of grids showing a significant positive correlation were 22.7%–86.7% (RWI or land NPP) and 6.9% (NDVI3g) (Table 3), whereas correlations with previous‐year data were much weaker for the ESM‐simulated land NPP.

Figure 6 shows the histograms for correlations of the RWI, NDVI3g, and land NPP with the summer precipitation for previous‐ and current‐year data. The RWI and NDVI3g had relatively similar patterns that were opposite to those shown in Figure 5. The correlations with the current‐ and previous‐year summer precipitation were more negative and positive, respectively. Among the grids, the RWI (NDVI3g) was considered herein; 17.7% (1.8%) and 8.2% (3.1%) showed significant positive and negative correlations with the previous‐ and current‐year summer precipitation, respectively (Tables 2, 3). The correlations between the land NPP (except ESM MPI) and current‐year summer precipitation were more positive than those for the RWI and NDVI3g, where the percentage of grids with significant positive correlations was 10.5%–40.5% (Table 3). The correlations between the ESM MPI and current‐year summer precipitation were more negative than those for the RWI, NDVI3g, and the other ESMs, in which 48.7% of the total grids had significant negative correlations (Table 3).

### 3.3 Density plots for the correlations of the RWI, NDVI3g, and land NPP with climate variables

**FIGURE 7:**
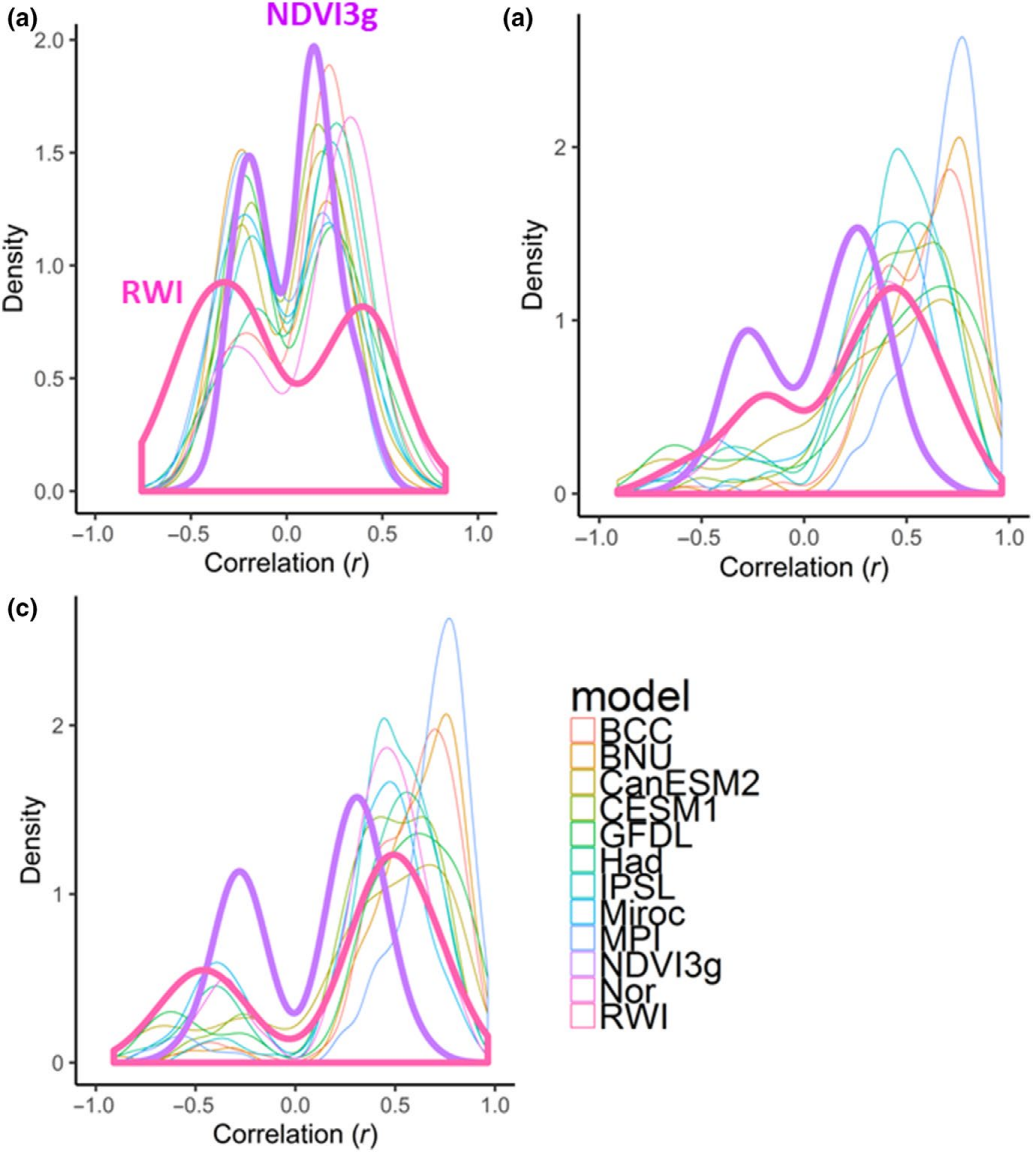
Density plots for correlations of the RWI, NDVI3g, and land NPP by ESMs with summer temperature or precipitation for (a) previous; (b) current; and (c) both years. The correlation coefficients were calculated based on meteorological data from both temperature and precipitation in the previous, current, and both years, respectively, for each grid with the highest correlation coefficient used for the density plot

Figure 7a shows density plots for the highest correlations of the RWI, NDVI3g, and land NPP with the summer temperature or precipitation for the previous‐year data. All plots had bimodal peaks with individual peaks on the positive and negative correlation sides. The plot shapes were very similar among the ESMs. In contrast, for the RWI, the peak of the negative correlation was biased toward being more significant than that of the ESMs and NDVI3g. The density plots for current‐year data are shown in Figure 7b. Similar to those for previous‐year data, the shapes of the plots were similar among the ESMs; however, the RWI and NEVI3g showed larger peaks for the negative correlation side, and peaks on the positive correlation side were biased toward being less significant than those for the ESMs. Figure 7c shows the density plots for both previous‐ and current‐year data. The correlation coefficients were calculated based on meteorological data for temperature and precipitation in both the previous and current years for each grid, and the highest correlation coefficient was then used for the density plot. The shapes were similar to those for the current‐year data, showing that the RWI and NDVI3g had larger peaks for the negative correlation side, and peaks on the positive correlation side were biased toward being less significant than those for the ESMs.

## 4 DISCUSSION

### 4.1 Responses of the RWI and NDVI3g to past climate change

The RWI and NDVI3g had clear opposite responses to summer temperature and precipitation between the previous‐ and current‐year data, which were never observed in the ESMs (Figures 5, 6). Both these indexes showed positive correlations with current summer temperatures and negative correlations with previous summer temperatures, although the RWI covered large areas with significant correlations (Figures 1 and 3). An opposite correlation pattern was observed for the summer precipitation (Figures 2 and 4). The observed negative response to summer temperatures and the positive response to precipitation likely indicate a reduction in the stomatal conductance of plants, which, under water‐limited conditions, would have resulted in lower plant carbon uptakes (Tei et al., 2014). Water availability is also closely coupled with cambial activity and the wood formation of trees (Balducci, Deslauriers, Giovannelli, Rossi, & Rathgeber, 2013), and radial growth requires the maintenance of high cell turgor pressure, which has an irreversible influence on cell extension and wall polymer deposition (Proseus & Boyer, 2005). In addition, warming‐induced carbon losses through plant respiration might have been the cause for the negative response.

The negative response was clearer for the RWI–temperature relationship than for the NDVI3g–temperature relationship (Figure 5a,b and Table 2) and was dominant within inner Alaska and Canada, central Europe, and eastern Siberia (Figure 1). This negative response of the RWI to warming corresponded with previous studies on local/regional tree‐ring and continental dry climate regions (Andreu‐Hayles et al., 2011; Barber et al., 2000; Silva, Anand, Oliveira, & Pillar, 2009; Tei, Sugimoto, Liang, et al., 2017; Tei, Sugimoto, Yonenobu, et al., 2017). A higher summer temperature could have caused a high evapotranspiration from surfaces, resulting in drier conditions that would have reduced the stomatal conductance in dry regions, which could have saved water losses by plant transpiration, but would also have reduced plant carbon uptakes (Tei et al., 2014).

Tei and Sugimoto (2018) reported smaller geographical areas of significant negative correlations with previous‐year summer temperatures for the NDVI3g than for the RWI, corroborating the results of this study (Figure 1a,b and Figure 5b, and Table 2). These authors suggested that satellite images could not reflect carbon losses through plant respiration, as is reflected in the RWI, because they only detect changes in leaf/needle greenness. However, even within inner Alaska and Canada, central Europe, and eastern Siberia, the NDVI3g did not have a dominant positive correlation; in fact, there was no significant correlation at all (Figure 1). Thus, in this sense, there was consistency with the results of the RWI.

The observed time lag responses of the RWI and NDVI3g to climate change (Figures 5, 6), i.e., negative and positive responses to summer temperature and precipitation, respectively, were also important features in the observation‐based indexes for forest response to climate change (Barber et al., 2000; Girardin et al., 2016; Tei & Sugimoto, 2018; Tei et al., 2014; Walker et al., 2015). Large differences in the RWI and NDVI3g responses to climate variables between the previous‐ and current‐year data were observed at lower (<500 mm) annual total precipitation sites (Figures S7–S8), suggesting that water‐stressed environments may be related to the observed time lag responses.

The adaptation of forest ecosystems to severely water‐stressed environments, i.e., carrying the carbon fixed during one particular year into the following year (Kagawa, Sugimoto, & Maximov, 2006; McDowell et al., 2008), could be the cause for this forest/tree response to climate change. Plants store fixed carbon as nonstructural carbon compounds, which are energy sources for biosynthesis in the following growing season (Chapin et al., 1990; Wiley & Helliker, 2012). Several studies have suggested that carbon storage could be an active process, occurring at the expense of superior growth during a particular year (Chapin et al., 1990; Genet et al., 2010) and could be a beneficial adaptive measure to severely water‐stressed environments. Increased carbon storage could reduce the risk of carbon starvation under severe drought conditions with reduced growth as a trade‐off (McDowell et al., 2008).

### 4.2 Different forest responses to past climate change in observation‐ and model‐based estimates

The simulated land NPP from most ESMs showed more significant correlations with climate variables in the current‐year data than that in the previous‐year data, with mostly positive correlations (Figures 5  and 6). The ESMs covered larger geographical areas (areal extent) and had more significant correlations with current summer climate variables (0.6%~86.7% for *p* < .05) (Table 3) than with previous summer climate variables (0.0%~13.8% for *p* < .05) (Table 2). These responses of the ESMs had a clearer relationship with annual average temperature than the RWI or NDVI3g (Figures S9 and S10). The simulated land NPP responses to the current‐year summer temperature changed from positive to negative as the annual average temperature increased (Figure S9), and the opposite change was observed for responses to the current‐year summer precipitation (Figure S10), suggesting that the land NPP responded negatively and positively to summer temperature and precipitation, respectively, in relatively high temperature regions. These responses were also observed in the RWI and NDVI3g; however, they were much weaker.

The observed overestimation of the simulated land NPP sensitivity to precipitation was applicable to all ESMs used in this study, except for ESM MPI, (Figure 6, Tables 2  and 3) as reported in previous RWI‐model studies (Rammig et al., 2015; Tei, Sugimoto, Liang, et al., 2017; Tei, Sugimoto, Yonenobu, et al., 2017). Some ESMs showed significant negative correlations with summer temperature within inner Canada, central Europe, and/or eastern Siberia, similar to the RWI, although this involved the current year and not previous year summer data (Figures 1 and 3, Tables 2  and  3). However, we clarified that the negative effect of summer temperature was masked by a much more critical contribution of summer precipitation for most of the simulated land NPP (Table 4). Therefore, the land NPP appeared to respond extremely positively to past temperature and precipitation changes compared with the RWI and NDVI3g (Figure 7b,c), both of which have been reported as RWI‐DGVMs climate sensitivity discrepancies in European forests (Klesse et al., 2018).

Thus, projections of the land NPP of circumboreal forests might be overestimated under the expected increases in both average global temperature and precipitation (IPCC, 2013). It would be difficult to estimate the extent of overestimation accurately; however, according to the observed dominated and significant negative response of the RWI to summer temperatures (Figure 1a), an overestimation is likely to be more pronounced in boreal forests than in the Arctic tundra and boundary ecosystem, especially for inland dry regions, such as inner Alaska and Canada, and eastern Siberia, and for hotter, southern regions, such as central Europe. In addition, the ESMs did not reproduce the time lag and the negative response to increased temperatures, which were observed in the RWI and NDVI3g (Figure 5). Especially in Alaska, no ESM reproduced the time lag and negative response (Figure 1).

These features for responses of the simulated land NPP to climate changes have been reported for SEIB‐DGVM (Sato et al., 2016) by Tei, Sugimoto, Yonenobu, et al. (2017) and for the ORCHIDEE and Lund–Potsdam–Jene models by Zhang et al. (2018). However, herein, we showed that these features could be applied to all current land carbon components used in the ESMs of CMIP5 over circumboreal forest ecosystems. Such discrepancies in observation‐model climate sensitivity have been well reported in European forests (Babst et al., 2013; Klesse et al., 2018; Zhang et al., 2018). It has been reported that the land NPP recovers faster after extreme climate events than the RWI (Anderegg et al., 2015; Rammig et al., 2015). No lag responses of the simulated land NPP were observed, compared to the RWI time lag responses to climate changes (Zhang et al., 2018). These previous studies suggested that the discrepancies in the observation‐model climate sensitivity might have resulted from inadequate model parameterization related to soil moisture availability and plant water potential (e.g. Huang, Gerber, Huang, & Lichstein, 2016; Rollinson et al., 2017), and/or from inadequate carbon allocation schemes. Klesse et al. (2018) especially pointed out that nonstructural carbohydrates, which are frequently recognized as adequate carbon reserve pools, are not well represented in the current version of ESMs, resulting in the models being highly sensitive to current‐year climate variables. Our results suggest that these observation‐model climate sensitivity discrepancies and related explanations could be applied to European and circumboreal forest ecosystems.

Both the NDVI3g and RWI are widely regarded as useful long‐term indicators of past forest ecosystem responses to climate change with reported differences in their responses (Buermann et al., 2014; Girardin et al., 2016; Tei & Sugimoto, 2018). However, our results indicated a different climate sensitivity for the RWI and NDVI3g, e.g., a weaker sensitivity of the NDVI3g to changes in summer temperature and precipitation in both previous and current years (Tables 2 and 3). The most likely reason for this discrepancy is that NDVI3g approaches could not reflect respiratory plant carbon losses because they only detect changes in leaf/needle greenness. Moreover, the observed large interannual variability in plant carbon allocation among organs and in the production of secondary plant compounds might also be a contributing factor (Litton, Raich, & Ryan, 2007). Although our results indicated the differences between the RWI and NDVI3g, these differences were smaller than those between observation‐ and model‐based estimates.

### 4.3 Future perspectives

We evaluated the land NPP simulated by the 10 ESMs involved in CMIP5 by comparing them with observation‐based indexes for forest productivity, namely NDVI3g and RWI, over circumboreal forests. We focused on forest responses to summer temperature and precipitation (Figures 1, 2, 3, 4 and Tables S1 and S2). However, climate variables other than the season have been found to affect vegetation activities over the northern high‐latitude regions (Tei & Sugimoto, 2018; Wang et al., 2011). Tei and Sugimoto (2018) reported that in the Arctic tundra and boundary ecosystems, the climate variables of the current summer were the main climatic drivers for the positive response to the increase in temperatures shown by both the NDVI3g and the RWI indices. In contrast, in boreal forest ecosystem, climate variables of the previous year (from summer to winter) were also important climatic drivers for both the NDVI3g and RWI. Importantly, both indices indicated that the temperatures in the previous year negatively affected the ecosystem. Such forest responses to climate variables other than the summer season were probably derived from changes in the timing of snowmelt in spring and/or the availability of nitrogen in the following summer (Kirdyanov, Hughes, Vaganov, Schweingruber, & Silkin, 2003; Sidorova et al., 2009; Tei, Sugimoto, Liang, et al., 2017; Vaganov, Hughes, Kirdyanov, Schweingruber, & Silkin, 1999). Tei and Sugimoto (2018) also reported that large geographical areas (areal extent) over circumboreal forests were significantly affected by temperature and precipitation in summer for both the RWI and NDVI3g. Nonetheless, the extent to which the spatial variability of seasonal climatic variables, controlling temporal variations in forest greenness (NDVI3g) and tree growth (RWI), over the northern high‐latitude regions could be reproduced by current ESMs is an important issue for further development of land carbon cycle components in ESMs.

In addition, the distribution of RWI sites in the International Tree‐Ring Data Bank is biased toward Europe and North America (Figures 1, 2, 3, 4 and Tables S1 and S2). Therefore, all datasets herein may have some regional bias, especially because we used only the NDVI3g and land NPP from grids over the RWI chronology sites. Further efforts to fill the gaps in RWI data, e.g., in central and western Canada and Siberia, are required. This study contributes to the understanding of RWI‐based forest responses to climate changes with fewer regional biases, resulting in a more useful validation dataset for simulations.

In summary, our results indicate that ESMs may overestimate the future forest NPP in continental dry regions under the expected increases in both average global summer temperature and precipitation, which are common features of current ESMs. Further efforts to evaluate simulated forest responses to climate changes in other seasons and to address the gaps in RWI data are important for improving empirical estimates, model structures, calibrations, and projections of ecosystem responses to climate changes over the northern high‐latitude regions.

## Supporting information

Supplementary Material
