# Supplementary material for: Excessive positive response of model‐simulated land net primary production to climate changes over circumboreal forests
Source: Plant Environ Interact. 2020 Jul 1;1(2):102–21. doi: 10.1002/pei3.10025 (PMC10168094; doi:10.1002/pei3.10025)
Supplement: Supplementary file 1 — Supplementary Material [file PEI3-1-102-s001.docx]

Supporting Information

Excessive positive response of model-simulated land net primary production

to climate changes over circumboreal forests

by Shunsuke Tei, Atsuko Sugimoto,

Fig. S1. Correlation coefficients of RWI (a) and land NPP by ESMs (c-l) with previous summer temperature for the period after 1950.

Fig. S2. Correlation coefficients of RWI (a) and land NPP by ESMs (c-l) with previous summer precipitation for the period after 1950.

Fig. S3. Correlation coefficients of RWI (a) and land NPP by ESMs (c-l) with current summer temperature for the period after 1950.

Fig. S4. Correlation coefficients of RWI (a) and land NPP by ESMs (c-l) with current summer precipitation for the period after 1950.

Fig. S5. Histogram for correlations of RWI (a) and land NPP by ESMs (c-l) with summer temperature for previous and current year for the period after 1950.

Fig. S6. Histogram for correlations of RWI (a) and land NPP by ESMs (c-l) with summer precipitation for previous and current year for the period after 1950.

Fig. S7. Correlations of (a) RWI; (b) NDVI3g; and (c–l) land NPP by ESMs with summer temperature for the previous and current year against annual total precipitation. Smooth local regressions are also shown.

Fig. S8. Correlations of (a) RWI; (b) NDVI3g; and (c–l) land NPP by ESMs with summer precipitation for the previous and current year against annual total precipitation. Smooth local regressions are also shown.

Fig. S9. Correlations of (a) RWI; (b) NDVI3g; and (c–l) land NPP by ESMs with summer temperature for the previous and current year against annual average temperature. Smooth local regressions are also shown.

Fig. S10. Correlations of (a) RWI; (b) NDVI3g; and (c–l) land NPP by ESMs with summer precipitation for the previous and current year against annual average temperature. Smooth local regressions are also shown.

Table S1 Tree-ring width chronologies on ITRDB

Table S1 Tree-ring width chronologies on ITRDB

Table S1 Tree-ring width chronologies on ITRDB

Table S1 Tree-ring width chronologies on ITRDB

Table S1 Tree-ring width chronologies on ITRDB

Table S1 Tree-ring width chronologies on ITRDB

Table S1 Tree-ring width chronologies on ITRDB

Table S1 Tree-ring width chronologies on ITRDB

Table S1 Tree-ring width chronologies on ITRDB

Table S1 Tree-ring width chronologies on ITRDB

Table S1 Tree-ring width chronologies on ITRDB

Table S1 Tree-ring width chronologies on ITRDB

Table S2 Tree-ring width chronologies from our observation sites

Reference

Axelson, J. N., Sauchyn, D. J., Barichivich, J., 2009. New reconstructions of streamflow variability in the South Saskatchewan River Basin from a network of tree ring chronologies, Alberta, Canada. Water Resources Research, 45, doi: 10.1029/2008WR007639

Barclay, D. J., Wiles, G. C., Calkin, P. E., 2003. An 850 year record of climate and fluctuations of the iceberg-calving Nellie Juan Glacier, south central Alaska, U.S.A. Annals of Glaciology, 36, 51-56.

Barclay, D. J., Barclay, J. L., Calkin, P. E., Wiles, G. C., 2006. A revised and extended Holocene glacial history of Icy Bay, southern Alaska, U.S.A. Arctic, Antarctic and Alpine Research, 38, 153-162.

Brown, D., Mike, B., 2005. “How old is that OAK?”in Ben Simon (ed.), A Treasured Landscape the Heritage of Belvoir Park. The Forest of Belfast, Belfast, 73-85.

Briffa, K. R., Melvin, T. M., Osborn, T. J., Hantemirov, R. M., Kirdyanov, A. V., Mazepa, V. S., Shiyatov, S. G., Esper, J., 2013. Reassessing the evidence for tree-growth and inferred temperature change during the Common Era in Yamalia, northwest Siberia. Quaternary Science Reviews, 72, 83-107, doi: 10.1016/j.quascirev.2013.04.008

Colenutt, M. E., Luckman, B. H., 1995. The dendrochronological characteristics of alpine Larch. Canadian Journal of Forest Research, 25, 777-789, doi: 10.1139/x95-085.

Colenutt, M., 2000. Climate reconstruction in the southern Canadian Rockies using tree-ring data from alpine larch. PhD dissertation, University of Western Ontario, London, Ontario.

Forbes, B. C., Fauria, M. M., Zetterberg, P., 2010. Russian Arctic warming and 'greening' are closely tracked by tundra shrub willows. Global Change Biology,16, 1542-1554, doi: 10.1111/j.1365-2486.2009.02047.x.

Friedrichs, D. A., Büntgen, U., Frank, D. C., Esper, J., Neuwirth, B., Löffler, J., 2009. Complex climate controls on 20th century oak growth in Central-West Germany. Tree Physiology, 29, 39-51, doi: 10.1093/treephys/tpn003.

Fischer, S., Neuwirth, B., 2012. Klimasensitivitat der Douglasie in Eifel und Kellerwald. Allgemeine Forst- und Jagdzeitung 183, 23-33.

Fischer, S., Neuwirth, B., 2013. Vulnerability of trees to climate events in temperate forests of west germany. ISRN Forestry, doi: 10.1155/2013/201360.

Gennaretti, F., Arseneault, D., Nicault, A., Perreault, L., Bégin, Y., 2014. Volcano-induced regime shifts in millennial tree-ring chronologies from northeastern North America. Proceedings of the National Academy of Sciences, doi: 10.1073/pnas.1324220111

George, S. S., Luckman, B. H., 2001. Extracting a paleotemperature record from Picea engelmannii tree-line sites in the central Canadian Rockies. Canadian Journal of Forest Research, 31, 457-470, doi: 10.1139/x00-188

Kaiser, K. F., 1993. Beiträge zur Klimageschichte ... Ziegler Druck- und Verlags-AG,

Winterthur, p.203.

Kenigsberg, M., 2005. The dendroclimatic potential of subalpine fir in the Canadian northwest. Unpublished M.Sc. Thesis, Department of Geography, The University of Western Ontario.

Kenigsberg, M. R., O’Reilly, B., Luckman, B. H., Moser, K. A., 2009. Lake sediment and tree-ring sampling in the Canadian Rockies, 2007-8. Report submitted to Parks Canada, Jasper, June 2009, p.23.

Jarvis, S. K., Wiles, G. C., Appleton, S. N., D'Arrigo, R. D., Lawson, D. E., 2013. A warming-induced biome shift detected in tree growth of Mountain Hemlock (Tsuga mertensiana (Bong.) Carriere) along the Gulf of Alaska. Arctic, Antarctic and Alpine Research, 45, 211-218, doi: 10.1657/1938-4246-45.211.

Luckman, B. H., Youngblut, D., 2000. Dendroclimatic investigations in the Southwest Yukon: A preliminary assessment. Report to Meteorological Service of Canada, Parks Canada and the Yukon Government, May 2000, p.52.

Luckman, B. H., Wilson, R. J. S., 2005. Summer temperatures in the Canadian Rockies during the last millennium: a revised record. Climate Dynamics, 24, 131-144, doi: 10.1007/s00382-004-0511-0

McComb, A., 2001. Anomolies in oak (Quercus petrare & Q. robur) tree rings and their relation to climate. MPhil thesis Queens University Belfast.

Myglan, V. S., 2010. Dendrochronological analysis of burials Churapcha region

(Republic of Sakha) // World Eurasia, pp.41-48. (in Russian, http://elibrary.ru/item.asp?id=18346247)

Neuwirth, B., Schweingruber, F. H., Winiger, M., 2007. Spatial patterns of central European pointer years from 1901 to 1971. Dendrochronologia, 24, 79-89, doi: 10.1016/j.dendro.2006.05.004.

O'Reilly, B., 2009. Dendroclimatological studies of Douglas fir (Pseudotsuga menziesii) at Jasper, Alberta, Canada. Unpublished B.Sc. Thesis, Department of Geography, University of Western Ontario.

Payne, M. 2006. The development of sitka spruce, white spruce and mountain hemlock ring-width chronologies from a transect along the Haines Road in the Yukon Territory and B.C. Unpublished B.Sc. Thesis, Department of Geography, University of Western Ontario.

Pederson, N., Leland, C., Nachin, B., Hessl, A. E., Bell, A. R., Martin-Benito, D., Saladyga, T., Suran, B., Brown, P. M., Davi, N. K., 2012. Three centuries of shifting hydroclimatic regimes across the Mongolian Breadbasket. Agricultural and Forest Meteorology, doi: 10.1016/j.agrformet.2012.07.003.

Perez-Valdivia, C., Sauchyn, D., 2011. Tree-ring reconstruction of groundwater levels in Alberta, Canada: Long term hydroclimatic variability. Dendrochronologia, 29, 41-47, doi: 10.1016/j.dendro.2010.09.001.

Sauchyn, D., Vanstone, J., Perez-Valdivia. C., 2011. Modes and Forcing of Hydroclimatic Variability in the Upper North Saskatchewan River Basin Since 1063. Canadian Water Resources Journal, 36, 205-217, doi: 10.4296/cwrj3603889.

Schultz, J. A., Neuwirth, B., 2012. A new atmospheric circulation tree-ring index (ACTI) derived from climate proxies: Procedure, results and applications. Agricultural and Forest Meteorology, 164, 149-160, doi: 10.1016/j.agrformet.2012.05.007

Tei, S., Sugimoto, A., Yonenobu, H., Ohta, T., Maximov, T.C., 2014. Growth and physiological responses of larch trees to climate changes deduced from tree-ring

widths and d13C at two forest sites in eastern Siberia. Polar Sci. 8, 183–195,

doi.org/10.1016/j.polar.2013.12.002.

Tei, S, Sugimoto, A, Liang, M., Yonenobu, H, Matsuura, Y, Oosawa, A, Sato, H, Fujunuma, J, Maximov, T. C., 2017. **Radial growth and physiological response of coniferous trees to Arctic amplification.** J. Geophys. Res, DOI: 10.1002/2016JG003745.

Tomkins, J.D., Lamoureux, S. F., Sauchyn, D. J., 2008. Reconstruction of climate and glacial history based on a comparison of varve and tree-ring records from Mirror Lake, Northwest Territories, Canada. Quaternary Science Reviews, 27, 1426–1441, doi: 10.1016/j.quascirev.2008.04.012

Youngblut, D. K., Luckman, B. H., 2013. Evaluating the temperature sensitivity of radial growth patterns from whitebark pine in the western Canadian Cordillera. Dendrochronologia, 31, 16-28, doi: 10.1016/j.dendro.2012.04.001

Wiles, G. C., Mennett, C., Jarvis, S. K., Lawson, D., D'Arrigo, R., 2012. Decline in Alaskan Yellow-Cedar: tree-ring investigations into climatic responses and possible causes: Glacier Bay, Alaska Canadian Journal of Forest Research, 42, 1-6, doi:10.1139/X2012-028

Wilson, R., Miles, D., Loader, N. J., Melvin, T., Cunningham, L., Cooper, R., Briffa. K., 2012. A millennial long March-July precipitation reconstruction for southern-central England. Climate Dynamics, 40, 997-1017, doi:10.1007/s00382-012-1318-z.
